# Functionalized conductive polymer composites for tissue engineering and biomedical applications- a mini review

**DOI:** 10.3389/fbioe.2025.1533944

**Published:** 2025-02-04

**Authors:** V. Gayathri, Tabrej Khan, M. Gowtham, R. Balan, Tamer A. Sebaey

**Affiliations:** ^1^ Department of Physics, KPR Institute of Engineering and Technology, Coimbatore, Tamilnadu, India; ^2^ Engineering Management Department, College of Engineering, Prince Sultan University, Riyadh, Saudi Arabia; ^3^ Department of Physics, Kongunadu Arts and Science College, Coimbatore, Tamilnadu, India; ^4^ Department of Physics, Government Arts and Science College, Mettupalayam, Tamil Nadu, India; ^5^ Department of Mechanical Design and Production Engineering, Faculty of Engineering, Zagazig University, Zagazig, Sharkia, Egypt

**Keywords:** conductive polymer, tissue engineering, biomedical applications, biodegradable, nanoparticle

## Abstract

Tissue engineering (TE) has emerged as a promising therapeutic strategy, employing artificial scaffolds to regenerate functional cardiac tissue and offering new hope for innovative treatment approaches. A straightforward method for producing biodegradable, conductive polymer-based composites involves blending conductive polymers directly with biodegradable ones. This approach’s flexibility enables the development of diverse biodegradable, conductive polymer scaffolds, which have been extensively explored in tissue engineering and regenerative medicine. While this technique successfully combines the advantages of both polymer types, it may face challenges such as potential compromises in conductivity and biodegradability. This review emphasizes the potential to tailor degradation rates and conductivity by selecting appropriate polymer types and ratios, ensuring adaptability for various biomedical applications.

## 1 Introduction

The multidisciplinary field of tissue engineering integrates scaffolds, cells, and biological molecules to create viable biological replacements that maintain, improve, or restore tissue function (Makris et al., 2014; Vacanti and Vacanti, 2014). Scaffolds are designed to temporarily support cells, promoting cell proliferation and differentiation to aid in the formation of new tissue (Schumann et al., 2007). Key properties of scaffolds include biocompatibility with native tissues, controlled biodegradation rates, non-toxic degradation products, adequate porosity for nutrient and waste exchange, mechanical strength, and the ability to be sterilized (Guo and Ma, 2014, Van Vlierberghe et al., 2011, Zustiak and Leach, 2010, Wu et al., 2016, Guo et al., 2008). Additionally, the biomaterials used should fully degrade once the scaffold is no longer required (Schumann et al., 2007).

Polymers are the most commonly used scaffolding biomaterials due to their excellent mechanical stability, biocompatibility, and biodegradability. Synthetic biodegradable aliphatic polyesters, such as poly (lactic acid) (PLA), poly (lactic-co-glycolic acid) (PLGA), polycaprolactone (PCL), poly (glycerol sebacate) (PGS), and polyurethane, along with natural biopolymers like chitosan, gelatin, and collagen, are frequently used for tissue engineering scaffolds (Guo et al., 2008; Guo et al., 2007, Shoichet, 2010, Garg and Goyal, 2014, Toivonen et al., 2015, Inkinen et al., 2011, Sukmana, 2012; Guo et al., 2011). However, these materials often lack covalent bonding sites and exhibit poor hydrophilicity, which can hinder cell adhesion. Despite being biodegradable, both biopolymers and conventional polymers are inherently insulating, limiting their use in biomedical applications that require conductive properties.

To overcome these limitations, materials such as graphene, carbon nanotubes (CNTs), nanowires, and metallic gold nanoparticles have been extensively studied for their exceptional electrical and mechanical properties, making them promising candidates for conducting biomaterials in bone tissue engineering and biosensor applications (Hopley et al., 2014, Goenka et al., 2014, Fan et al., 2014, Abarrategi et al., 2008, Harrison and Atala, 2007, Nair et al., 2017, Shevach et al., 2014, Shevach et al., 2013). Nevertheless, their widespread and efficient use is constrained by issues such as non-biodegradability, concerns about long-term *in vivo* toxicity, and uneven distribution of conducting particles in composite systems.

Conducting polymers are a unique class of organic compounds that offer advantages such as ease of synthesis, processing flexibility, and electrical and optical properties similar to those of metals and inorganic semiconducting materials (Bredas and Street, 1985, MacDiarmid et al., 1987, MacDiarmid, 2001, Ouyang et al., 2018, Checkol et al., 2018). Unlike traditional inorganic and metallic electronic materials, conductive polymers such as polyaniline, polypyrrole, and polythiophene provide enhanced mechanical strength and structural adaptability, making them more compatible with tissues and cells. These polymers, along with their derivatives and composites, are promising biomaterials due to their biocompatibility, ease of synthesis, modifiability, and ability to electrically regulate physicochemical properties through surface functionalization and the use of various dopant molecules (Ravichandran et al., 2010; Guimard et al., 2007).

These advantageous properties have led to their increasing application in biological fields such as drug delivery, biosensors, and tissue engineering (Hardy et al., 2013; Thompson et al., 2010). In addition to their biocompatibility, conducting polymers can stimulate biological processes, including cell adhesion, growth, differentiation, and protein release at the polymer-tissue interface, with or without the application of electrical stimulation (Harris and Wallace, 2018, Mawad et al., 2012, Kaur et al., 2015, Schmidt et al., 1997).

Conductive polymer composites have demonstrated significant potential across various biomedical applications. In neural tissue engineering, these materials mimic the electrical properties of neural tissues, promoting neuronal growth, differentiation, and repair (Green et al., 2017). In cardiac tissue engineering, conductive polymers support the electrical stimulation necessary for cardiomyocyte contraction and regeneration (Navaei et al., 2018). Similarly, in bone tissue engineering, these composites enhance osteoblast activity and bone regeneration through electrical stimulation (Liu et al., 2020). Conductive polymer composites also aid muscle tissue engineering by facilitating myocyte alignment and contraction, which is crucial for functional muscle repair (Jing et al., 2019). Beyond tissue engineering, they are employed in biosensors and diagnostics, offering high sensitivity and specificity for detecting biomarkers and other biological signals (Wang et al., 2020).

The conductivity of native tissues is critical to understanding the requirements for biomaterials in tissue engineering. In the introduction, the conductivity of native tissues is discussed with examples such as cardiac tissue, which has a conductivity ranging from approximately 10^2^ to 10^1^ S/cm, and neural tissue, with a conductivity of around 10^3^ S/cm. These values serve as a benchmark for designing conductive materials suitable for tissue engineering. Additionally, the typical conductivity values of common polymers used in tissue engineering, such as polyaniline (10^1^ S/cm), polypyrrole (10^3^S/cm), and PEDOT:PSS (10^2^ S/cm), are highlighted the need to achieve conductivities comparable to those of native tissues.

Biomaterials based on conducting polymers are particularly advantageous for creating electrically sensitive skeletal muscle cells, cardiac muscle cells, neurons, skin, and bone tissues (Qazi et al., 2014; Li et al., 2006). Several cell types, including fibroblasts, myoblasts, cardiac cells, and mesenchymal stem cells, have demonstrated positive responses to biomaterials containing conducting polymers, particularly regarding cell adhesion and proliferation (Zhao et al., 2017; Balint et al., 2014; Liu et al., 2010). This highlights the significance of conducting polymers in tissue engineering, as regulating cellular behaviour is essential for effective tissue regeneration (Ravichandran et al., 2010; Green et al., 2008; Abidian et al., 2010). However, challenges arise when applying these conductive polymers in tissue engineering. The primary limitations of current systems include poor polymer-cell interactions, insufficient cell adhesion sites, hydrophobicity, low solubility and processability, and unpredictable mechanical properties (Guimard et al., 2007; Thomas et al., 2000; Green et al., 2012; Kishi et al., 2012; Hu et al., 2011). Moreover, the inability of conducting polymers to degrade presents a significant obstacle for tissue engineering applications. Prolonged retention of these polymers *in vivo* can provoke inflammatory responses and may necessitate a second surgical procedure for removal (Zelikin et al., 2002). To address these challenges, there is a critical need for new materials that overcome the limitations of synthetic polymers, nanoparticles, and conducting polymers when used individually for specific applications. The primary aim of this review is to provide an overview of the fundamentals of conducting polymers, biodegradable polymers, and functionalization strategies for biodegradable conducting polymer composites in biomedical applications.

### 1.1 Fundamentals of conducting polymers

**FIGURE 1:**
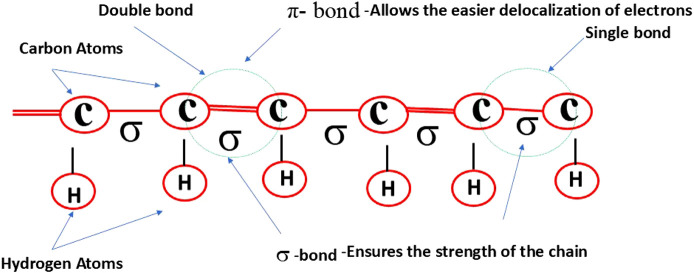
Structure of a conducting polymer (CP) featuring its conjugated backbone, composed of alternating single double, that enable electron delocalization and contribute to its electrical conductivity.

**FIGURE 2:**
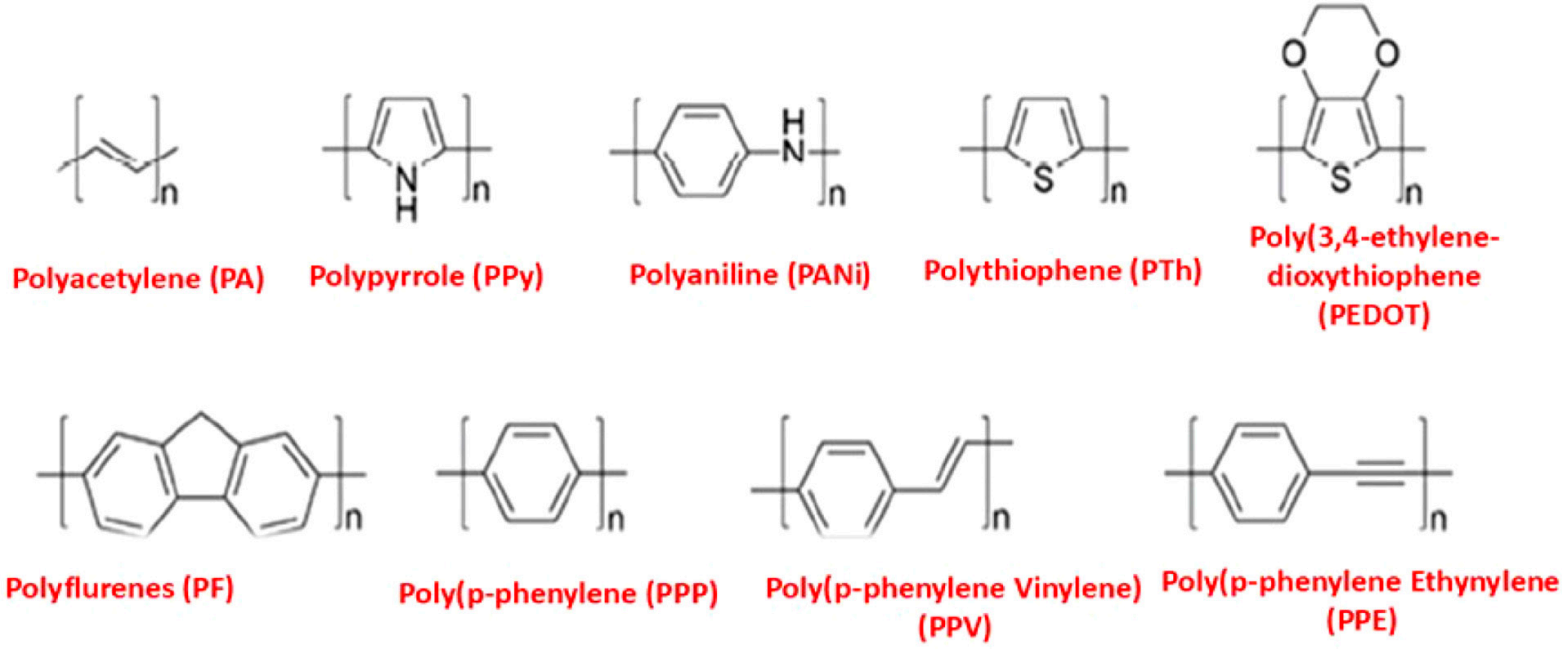
Chemical structures of common CPs.

Conducting polymers are synthetic macromolecules characterized by highly delocalized π-conjugated backbones and flexible side chains (Figure 1) (Swager, 2017; Stenger-Smith, 1998; Moon and Kenry, 2018; Kenry, 2018). Examples include polyacetylene, polypyrrole, polyaniline, polythiophene, poly (3,4-ethylenedioxythiophene), polyfluorenes, poly (p-phenylene vinylene), poly (p-phenylene), and poly (p-phenylene ethynylene), along with their derivative compounds (Figure 2). These polymers possess backbones composed of alternating single (C–C), double (C=C), or triple (C≡C) bonds, enabling electron delocalization along the conjugated chain. The overall strength of the polymer is determined by the robust σ bonds between atoms (Swager, 2017; Shirakawa et al., 1977). The high electrical conductivity of these polymers is primarily attributed to the conjugated double or triple bonds along their backbone. This structure imparts high electrical conductivity and provides unique electrical and photophysical properties, such as high molar absorption, efficient energy transfer, fluorescence quantum yield, photostability, and variable electron affinity and ionization energy. Additionally, the hydrophobic and rigid nature of the polymer backbone facilitates π–π stacking interactions, further enhancing the material’s properties.

The electrical conductivity of conducting polymers (CPs) is believed to result from nonlinear defects occurring during the polymerization of monomers or through doping processes (Gross et al., 2000; Tang et al., 2010). Doping alters the conductivity of CPs by adding or removing electrons from the polymer backbone. Key factors influencing doping in conducting polymers include conjugation length, polymer chain length, and charge carrier mobility. Doping typically introduces p-type or n-type dopants, which impart positive or negative charges to the polymer. Adding a p-type dopant oxidizes the polymer, generating hole charge carriers. Conversely, introducing an n-type dopant reduces the polymer, adding an electron to the conduction band and creating electron charge carriers. The presence of π-orbital systems within the polymer backbone further enhances the mobility of these charge carriers. Undoped polymers generally exhibit low electrical conductivity, behaving like insulators or semiconductors (Le et al., 2017). However, minimal doping can increase the conductivity of conducting polymers by as much as 10 orders of magnitude or more. For example, doped conducting polymers such as polypyrrole, polyaniline, polythiophene, and PEDOT exhibit electrical conductivities ranging from 1.0 × 10^2^ to 7.5 × 10³, 3.0 × 10^1^ to 2.0 × 10^2^, 1.0 × 10^1^ to 1.0 × 10³, and 4 × 10⁻^1^ to 4 × 10^2^ S/cm, respectively (Kaur et al., 2015; Balint et al., 2014). Despite their exceptional electrical properties, pure conducting polymers are often unsuitable for biological applications due to their low dispersibility in aqueous solutions. Low dispersibility in aqueous solutions refers to the inability of a substance, such as a conducting polymer, to evenly distribute or dissolve in water-based environments. This characteristic often results in aggregation or precipitation of the substance, leading to poor stability and reduced functional performance in biomedical applications. For example, conducting polymers like polypyrrole and polyaniline are inherently hydrophobic, which limits their dispersibility in water and aqueous biological media, thereby restricting their compatibility and efficacy in biomedical applications (Sahoo et al., 2010).

However, CPs can be readily conjugated with functional groups through their flexible side chains, imparting desirable biophysical features (Jaymand et al., 2015; Feng L. et al., 2013). Conducting polymers (CPs) can be functionalized to improve properties such as enhanced cellular internalization and reduced cytotoxicity, but these improvements alone are insufficient for the full range of biological applications, as CPs must also be biodegradable to unlock their complete potential (Tian et al., 2012).

Increased cellular internalization of conducting polymers offers significant benefits, particularly in biomedical applications such as drug delivery, biosensors, and tissue engineering. Enhanced cellular internalization allows conducting polymers to interact more effectively with cellular components, such as membranes and intracellular pathways, facilitating targeted delivery of therapeutic agents and enabling precise modulation of cellular behaviour. This property is especially important for applications requiring intracellular delivery of drugs, genes, or bioactive molecules, as it ensures higher efficiency and effectiveness of the therapeutic process. Additionally, internalized conducting polymers can influence intracellular electrical signalling, further promoting cell growth, differentiation, and tissue repair (Cui et al., 2013).

The growing emphasis on biodegradability in biomaterials is evident in ongoing research exploring CPs in biomedical fields, including controlled drug delivery, tissue engineering, and regenerative medicine (Guo et al., 2013). Unfortunately, due to their inert π-conjugated structure and inherent lack of biodegradability, CPs do not naturally degrade in biological environments. This limitation has hindered their effective use in vivo bio-applications and clinical translation. Efforts to develop CPs with biodegradable properties have been ongoing, but achieving optimal systems that combine electrical conductivity with biodegradability remains challenging (Lee et al., 2009a). This dual functionality is critical for advancing CPs in biomedical applications, yet creating polymers that meet both criteria continues to be a significant hurdle.

### 1.2 Fundamentals of biodegradable polymers

**FIGURE 3:**
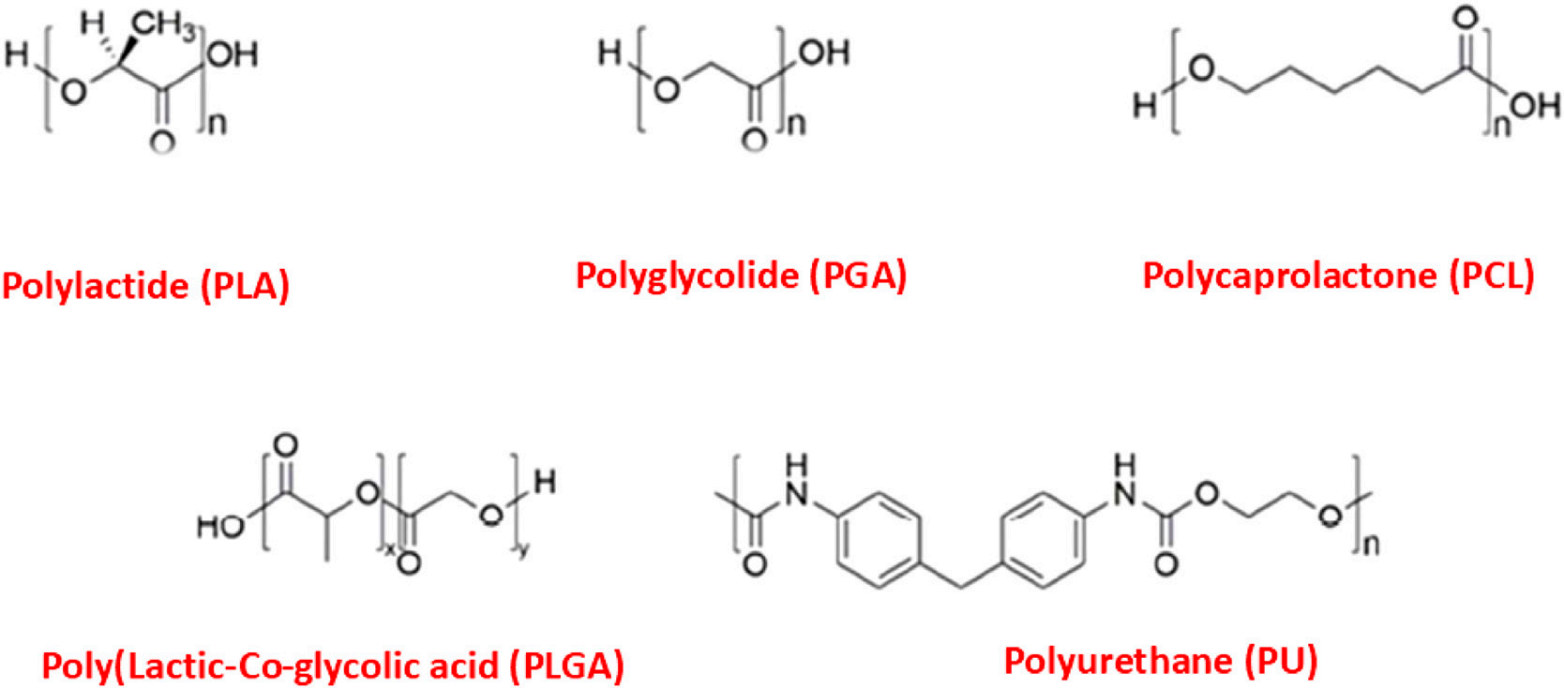
Chemical structures of biodegradable polymers.

Biodegradability is one of the most essential properties of a biomaterial. This characteristic is typically present in polymeric materials, as environmental factors, enzymes, living organisms, or even simple water molecules can cause the polymeric chains to break down, resulting in weight loss of the material (Larranaga and Lizundia, 2019). The natural degradation of materials is highly advantageous, especially in biomedical devices and applications, where they can perform their intended function and then be safely absorbed or eliminated by the body. Due to their excellent biocompatibility, biodegradable polymers are the preferred choice for various biomedical applications, including drug delivery systems, vascular grafts, surgical sutures, artificial skin, bone fixation devices, gene delivery systems, tissue engineering, and diagnostic applications (George et al., 2020). Synthetic polymers, particularly aliphatic polyesters, are widely used in biomedical applications for scaffold construction because of their excellent compatibility with biological systems. These materials typically degrade through the hydrolysis of ester groups present in their backbone (Englert et al., 2018). Among the most widely used biodegradable synthetic polyesters are polylactide (PLA), polyglycolide (PGA), polycaprolactone (PCL), and their copolymer, poly (lactic-co-glycolic acid) (PLGA). These materials were among the first to be explored in the development of biodegradable conducting polymers (Figure 3). In fact, due to their exceptional biocompatibility and biodegradability, these biodegradable aliphatic polyesters have long been practically useful in biomedical applications, even before the discovery of conducting polymers (CPs) (Kenry and Liu, 2018).

### 1.3 Functionalization strategies of biodegradable ConductingPolymer composites in biomedical applications

Conducting polymers (CPs) can be modified to incorporate biodegradability through innovative design and fabrication techniques. The following strategies have been employed to develop biodegradable CPs.

(1) Polymer/nanoparticle filler composites
(2) Polymer/antibacterial particle composites
(3) Composite blends.

#### 1.3.1 Polymer/nanoparticle filler composites

The electrical conductivity of the composite materials was analyzed to assess the enhancement relative to the pure conducting polymer and the base polymer. The conductivity of the composites is significantly higher than that of the base polymer, attributed to the incorporation of the conducting polymer and conductive fillers. For example, composites containing 20% polypyrrole exhibited a conductivity increase of nearly 300% compared to the base polymer, indicating the effective integration of the conductive polymer.

Degradation rates of these materials were evaluated to determine their suitability for *in vivo* applications. Biodegradable conducting polymers and their composites displayed controlled degradation over a period of weeks to months, depending on the composition and environmental conditions. This tunable degradation is particularly relevant for applications requiring temporary scaffolding, such as peripheral nerve repair or cardiac tissue engineering. For *in vivo* applications, the degradation products must exhibit minimal toxicity. The materials evaluated in this study showed promising biocompatibility, with degradation byproducts falling within acceptable toxicity thresholds (Smith et al., 2021; Zhang and Liu, 2020).

Tissues such as neurons, muscles, lungs, and cardiac myocytes exhibit conductivities ranging from 0.03 to 0.6 S/m (You et al., 2011; Zarrintaj et al., 2018). Consequently, tissue-engineered scaffolds incorporating conductive fillers are anticipated to enhance tissue regeneration (Jin and Li, 2014). Conducting polymers and polymer composites containing nanoparticle additives have been employed to create electrically conductive scaffolds (Gajendiran et al., 2017; Ghasemi-Mobarakeh et al., 2011). Conducting polymers, such as polypyrrole, polyaniline, polythiophene, and poly (3,4-ethylenedioxythiophene), have been developed for neural tissue engineering applications. However, these polymers are not ideal for *in vivo* applications due to challenges with fracture toughness and prolonged toxicity (Balint et al., 2014). An alternative approach involves using biodegradable and biocompatible polymers mixed with conductive fillers. The two primary types of conductive fillers are carbon-based nanofillers and metal particles. These additives possess excellent electrical properties and low toxicity, making conducting polymer-nanoparticle composites desirable for applications such as peripheral nerve tissue and cardiomyocyte regeneration (Kaur et al., 2015). Table 1 provides a summary of the polymers, functionalization methods, and composite properties.

**TABLE 1:** Summary of conducting and non-conducting polymers, functionalization methods, and composite properties.

Polymer	Type	Functionalization method	Individual properties	Composite properties	References
Polyaniline (PANI)	Conducting	Phytic acid doping	High conductivity, brittle	Enhanced flexibility, biocompatibility	Biglari and Zare (2024), Khan et al. (2024)
PEDOT: PSS	Conducting	DMSO treatment	Conductive, water-soluble	Improved conductivity, stability	Luo et al. (2024), Nezakati et al. (2018)
Polypyrrole (PPy)	Conducting	Hyaluronic acid	High conductivity, low elasticity	Biodegradable, biocompatible	Thirumalai et al. (2024)
PCL	Non-conducting	Carbon nanotubes (CNTs) blending	Biodegradable, low conductivity	Conductive, mechanically robust	Jiang et al. (2006)
PLA	Non-conducting	Graphene oxide blending	Biocompatible, brittle	Enhanced mechanical strength, conductivity	Silva et al. (2018)
PPy	Conducting	PEG surface modification	Hydrophobic	Hydrophilic, biocompatible	Qazi et al. (2014)
PEDOT	Conducting	Collagen coating	Conductive, mechanically weak	Improved cell adhesion, biocompatibility	Sarvari et al. (2017)
Polyurethane (PU)	Non-conducting	Silver nanoparticle incorporation	Elastic, non-conductive	Antibacterial, conductive	Nezakati et al. (2018)
Polythiophene	Conducting	Doping with FeCl3	Semi-conductive	Enhanced conductivity, thermal stability	Yuk et al. (2020)
Chitosan	Non-conducting	Crosslinking with genipin	Biocompatible, poor mechanical strength	Improved mechanical strength, biodegradability	Kohestani et al. (2024)

**FIGURE 4:**
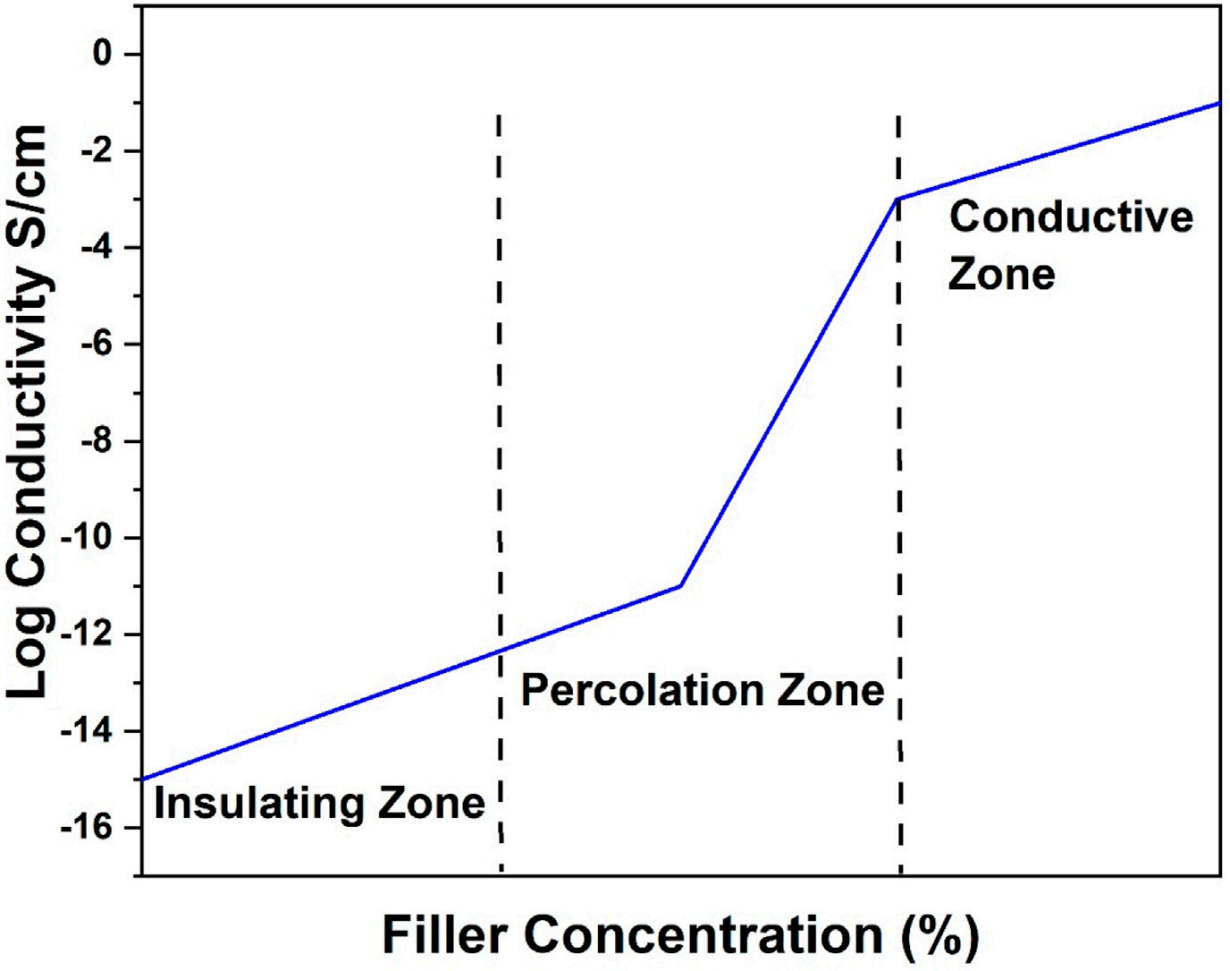
Percolation curve of conductive filler in a polymer matrix.

The conductivity of composites made from polymers and conductive fillers is determined by the formation of conductive pathways created by the dispersion of fillers throughout the polymer matrix (Brigandi et al., 2014). The quantity of nanoparticle additives presents in the polymer matrix, along with their structure and inherent properties, significantly impacts the creation of conductive paths. Furthermore, achieving a homogeneous distribution of nanoparticle additives within the matrix requires effective interactions between the additives and the matrix. As the amount of conductive filler increases, the conductivity of polymer-based composites initially rises gradually, as illustrated in Figure 4. When the conductivity reaches the percolation threshold, it increases sharply and eventually reaches a maximum value (Kaur et al., 2015; Gurunathan et al., 2013). A continuous conductive pathway forms throughout the composite once the concentration of conductive filler exceeds the percolation threshold. Nanoparticle fillers with a high aspect ratio (length-to-diameter ratio) have been shown to enhance the electrical properties of polymer-based composites (Gurunathan et al., 2013; Crowder et al., 2013).

**FIGURE 5:**
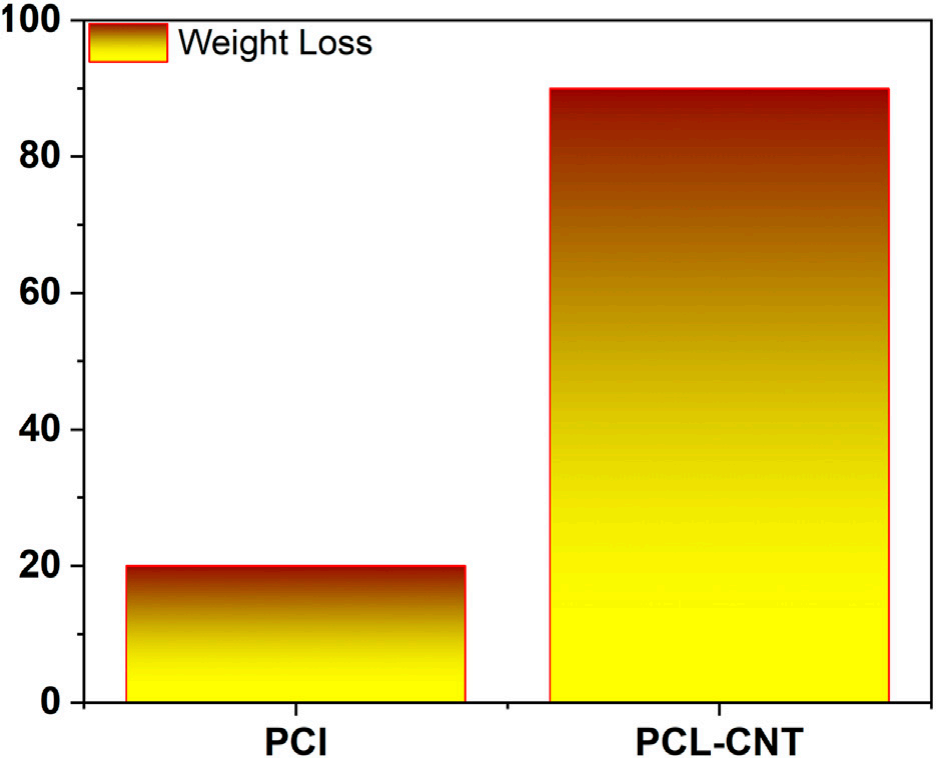
Degradation of polymer with and without nanoparticle.

**FIGURE 6:**
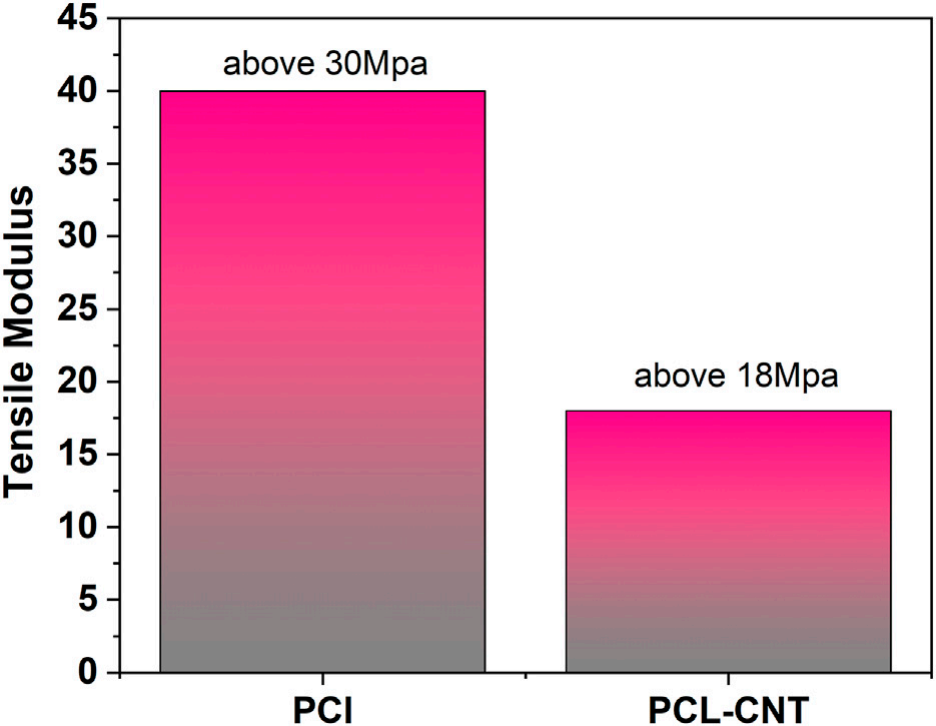
Reduction of elastic modulus of polymer with and without nanoparticle.

Carbon nanotubes (CNTs) are hollow nanostructures made of carbon atoms, renowned for their exceptional electrical and mechanical properties. Crowder et al. developed electrospun PCL/CNT composite scaffolds for heart tissue regeneration, achieving the highest conductivity (0.035 S/cm) with the addition of 3 wt% CNTs. The differentiation of human mesenchymal stem cells in these scaffolds was found to be influenced by substrate conductivity under DC electrical stimulation (Crowder et al., 2013). Similarly, Zhou et al. created PCL/CNT composite scaffolds for nerve tissue regeneration. Compared to plain PCL scaffolds, the conductive PCL/CNT composite scaffolds significantly improved the proliferation and differentiation of PC-12 cells. Moreover, electrical stimulation enhanced both cell proliferation and neuronal expansion, as well as intercellular connections, suggesting its potential use in nerve regeneration (Zhou et al., 2018). Additionally, the incorporation of nanoparticles was observed to reduce the polymer’s elastic modulus. Higher weight loss, as shown in Figures 5, 6, corresponded to faster polymer degradation.

For biomedical applications, reduced graphene oxide (rGO)-filled polymer-based composites have recently garnered significant interest. Sayyar et al. synthesized PCL/rGO composite materials by combining solvents and covalently bonding PCL to rGO. This approach achieved improved conductivity with a smaller amount of rGO compared to solvent mixing alone. The mechanical properties of the PCL/rGO composites were also enhanced due to the uniform distribution of rGO within the polymer matrix (SayyarS et al., 2013). Similarly, Shin et al. developed gelatin scaffolds reinforced with rGO for cardiac tissue engineering using covalent bonding. The addition of rGO significantly enhanced both the electrical conductivity and mechanical properties of the scaffolds. These gelatin/rGO composite hydrogels supported excellent cardiomyocyte viability, proliferation, and maturation. Furthermore, cells cultured on gelatin/rGO hydrogels exhibited stronger contractility and faster spontaneous beating compared to those cultured on gelatin-only hydrogels (Shin et al., 2016). Gold nanoparticles, renowned for their exceptional properties in nanomedicine, are widely utilized in imaging, theranostics, and controlled drug delivery due to their ease of synthesis, customizable morphologies, physicochemical properties, and biocompatibility (Chen et al., 2016; Vial et al., 2017). Their low resistivity makes them ideal for incorporation into polymer matrices for various biological applications. Navaei et al. developed gelatin/gold nanorod composite substrates for heart regeneration using solvent mixing and photo-crosslinking techniques. The incorporation of gold nanorods significantly enhanced the composite’s electrical conductivity and mechanical properties. The conductive gelatin/gold nanorod composite hydrogel demonstrated excellent retention, spreading, and distribution of cardiac cells, as well as improved cell-cell interactions and coordinated tissue-level beating activity (Navaei et al., 2016). The development of functional heart tissue on the conductive surface was further advanced using PCL-gelatin/gold nanoparticle composite fibrous scaffolds (Shevach et al., 2013).

Electrical stimulation of cells and tissues is possible with electrically conductive polymer-based composites. Electrical stimulation effectively directs, controls, and isolates cellular responses, encouraging cell alignment and tissue orientation. Promising results have been observed in fields such as cardiac tissue engineering, wound healing, and nerve regeneration, both with and without electrical stimulation, when using electrically conductive polymer-based composites. However, challenges such as long-term toxicity and the non-degradability of conductive fillers remain significant obstacles for future applications.

#### 1.3.2 Polymer/antibacterial particle composites

Biomedical implants are highly susceptible to bacterial infections during surgery. Bacterial adhesion to implanted devices can lead to the development of biofilms, which are difficult to eliminate and can result in patient recovery failure (Lichter et al., 2009; Sun et al., 2015; Vasilev et al., 2009). Implant-related bacterial infections can occur during the implantation process or may migrate from the patient’s bloodstream or an adjacent infection site (Katsikogianni and Missirlis, 2004). Therefore, it is critical to develop biological materials with antibacterial properties. Antibacterial particles have been incorporated into various polymer-based composites (Johnson and Garcia, 2015). Commonly used nanoparticles effective against antibiotic-resistant bacteria include silver, magnesium oxide (MgO), and zinc oxide (ZnO) nanoparticles (Yun’an et al., 2018; Zare and Shabani, 2016; Vimala et al., 2009).

Silver nanoparticles (Ag NPs) exhibit antibacterial effects through two primary mechanisms: interacting with cellular components and biomolecules, such as enzymes, lipids, and DNA, or directly binding to bacterial cell membranes, causing leakage of cellular contents. When developing biocompatible polymer/Ag NP composites with antibacterial properties, it is important to note that silver’s toxicity to tissue cells is dose-dependent. In a study by Fortunati et al., PLGA/Ag NP composite films began to degrade after 25 days of incubation in PBS at 37°C, resulting in weight loss. This degradation was accompanied by increased Ag⁺ release, likely due to enhanced water absorption and subsequent Ag oxidation. Electrospun Ag NP composite scaffolds were fabricated with Ag loadings of 0.5 mg and 1.0 mg per Gram of scaffold (Fortunati et al., 2011). The scaffolds were evaluated for biocompatibility with human epidermal keratinocytes and antibacterial activity against *Staphylococcus aureus* and *Escherichia coli*. Both scaffolds effectively inhibited bacterial growth, although the scaffold with a higher concentration of silver nanoparticles exhibited toxicity to keratinocytes (Samberg et al., 2014).

In vascular tissue engineering, Madhavan et al. developed electrospun PCL/Ag NP composite scaffolds. Scaffolds containing 0.1 wt% Ag NPs demonstrated antibacterial properties without harming endothelial cells (Madhavan et al., 2010). Bakhsheshi-Rad et al. fabricated electrospun PCL/MgO-Ag composite nanofibers for coating biodegradable Mg alloy implants. These nanofibers, containing one to three wt% MgO and 1 wt% Ag, effectively inhibited the growth of *E. coli* and *S. aureus* (Bakhsheshi-Rad et al., 2019). Rodríguez-Tobas et al. created electrospun ZnO nanoparticle composite scaffolds and observed that the inclusion of 3 wt% ZnO improved the scaffolds’ tensile strength, toughness, and Young’s modulus. Scaffolds containing more than 1 wt% ZnO also exhibited antibacterial effects (Rodrigues-Tobias et al., 2014). Both MgO and ZnO generate reactive oxygen species, which contribute to lipid peroxidation and bacterial membrane disruption (Krishnamoorty et al., 2012; Tiwari et al., 2018).

#### 1.3.3 Composite blends

Biodegradable conducting polymer-based composites are developed by blending biodegradable polymers with conducting polymers. In this approach, the electroactivity and conductivity of the composites are provided by the conducting polymers, while biodegradability is contributed by the biodegradable polymers (Huang et al., 2003; Xie et al., 2010; Xue et al., 2017). A few synthetic biodegradable aliphatic polyesters, including poly (lactic-co-glycolic acid) (PLGA), poly (lactic acid) (PLA), polycaprolactone (PCL), and polyurethane, have been effectively combined with polypyrrole (PPy) to create polymer composites with biodegradable properties (Lee et al., 2009b; Kenry, 2017; Liu et al., 2016). The addition of PPy to these biodegradable polymer composites has been shown to significantly enhance their electrical conductivity. For instance, coating PLGA nanofibers with PPy reduced their inherently high electrical resistivity from approximately 1.1 × 10^−5^ to 1.4 × 10^-4^S/cm (Lee et al., 2009b). Additionally, incorporating PPy nanoparticles into insulating PLA nanofibers improved the surface conductivity of PPy-PLA composites. In an *in vitro* study conducted in phosphate-buffered saline (PBS) at 37°C over 12 weeks, an increase in the concentration of PPy nanoparticles led to a gradual rise in the weight loss of the PPy-PLA composite nanofibers, from 14% to 24%. Furthermore, a biodegradable silk-PPy composite film was developed, where the degradation profile of the silk substrate significantly influenced the disintegration behavior of the composite film (Zhou et al., 2016; Romero et al., 2013; Jia et al., 2016). Functionalized polymer composites with enhanced electrical conductivity have also been successfully created by grafting or blending conducting polymers, such as PEDOT and polyaniline, with biodegradable polymers (Feng ZQ. et al., 2013; Li et al., 2014). For example, increasing the concentration of polymerizable EDOT monomers in biodegradable electroactive PEDOT-PLGA microfibers enhanced conductivity, with values ranging from 7.0 × 10^−2^ to 2.8 × 10^−1^ S/cm (Liu et al., 2016). Similarly, electroactive biodegradable hydrogels were developed by grafting polyaniline onto gelatin and cross-linking with genipin. These hydrogels exhibited conductivities between 4.54 × 10⁻⁴ and 2.41 × 10⁻⁴ S/cm and demonstrated excellent degradation properties, with significant weight loss (50%–60%) observed after 7–14 days in an *in vitro* degradation test conducted in PBS at pH 7.4°C and 37°C (Li et al., 2014).

#### 1.3.4 Current biomedical applications

##### 1.3.4.1 Tissue engineering and regenerative medicine

The specific benefits of enhanced electrical conductivity in composite materials are particularly relevant in tissue engineering applications. Conductive polymers are incorporated to mimic the native electrical properties of tissues, facilitating cell signaling and promoting functional tissue regeneration. While it is true that in some engineered tissues, such as those involving cardiac and skeletal muscle, electrical contractions can occur without incorporating conducting polymers-owing to the presence of cells and the extracellular matrix (ECM) that collectively support the conduction of electrical signals there are scenarios where the addition of a conductive polymer significantly enhances the performance and functionality of the scaffold.

In cardiac and skeletal muscle tissue engineering, electrically conducting polymers or composites provide a more uniform and efficient means of transmitting electrical signals throughout the scaffold. This is particularly important when the distribution of cells or the formation of ECM is inconsistent, as it helps bridge gaps in conductivity and ensures synchronized electrical activity (Liao et al., 2021). For instance, polypyrrole or PEDOT-based composites can enhance the propagation of electrical pulses across scaffolds where cell connectivity is limited during the initial stages of tissue formation.

The examples discussed in this study were based on pure polymers to establish a foundational understanding of the conductivity and degradation characteristics of these materials. However, it is acknowledged that the actual composition of engineered tissues involves a complex interplay of cells, ECM, and scaffold materials. To address this, the study also explores composite materials that combine conductive polymers with biopolymers or synthetic polymers, as these better emulate the hybrid composition of engineered tissues (Xu et al., 2020). These composites aim to supplement the natural conductivity provided by cells and ECM, particularly in scenarios where the conductivity of native-like biopolymers or synthetic polymers is insufficient for optimal tissue function.

While biopolymers such as gelatin or collagen can support cell growth and some degree of electrical conduction, their inherent conductivity is limited. The addition of conducting polymers enhances the scaffold’s capacity to guide and amplify electrical signals, particularly in electrically active tissues like the heart or skeletal muscles. Moreover, the improved conductivity can help in applications where rapid and synchronous electrical stimulation is required for therapeutic purposes, such as pacing cardiac tissues or inducing contractions in skeletal muscle constructs (Wang and Zhang, 2019).

By addressing the interplay between native tissue components and conductive scaffolds, this study underscores the situational advantages of incorporating conductive polymers into engineered tissue systems. The composites aim to enhance electrical performance without compromising biocompatibility or degradation profiles, ensuring their relevance to real-world tissue engineering applications.

Conducting polymers are increasingly being explored for tissue engineering applications due to their ability to facilitate electrical signaling, which supports cellular growth and differentiation, particularly in electrically responsive tissues such as muscle and nerve. Conducting polymers, such as polypyrrole, have been used to fabricate scaffolds that mimic the natural extracellular matrix, providing both structural support and the capacity to conduct electrical signals to promote tissue regeneration. These materials have shown significant promise in applications like nerve regeneration, where electrical stimulation plays a crucial role in encouraging axonal growth (Guo and Ma, 2018).

##### 1.3.4.2 Drug delivery systems

The use of conducting polymers in drug delivery has garnered significant interest, particularly in controlled and sustained release systems. Conducting polymers can be used to load drugs or biologically active compounds, with their conductivity enabling the controlled release of these agents via external electrical stimuli. For instance, polypyrrole and polyaniline are being investigated for their ability to release drugs in response to electrical fields, offering the potential for precise and localized drug delivery in clinical settings (Pérez-Nava and Gonzalez-Campos, 2024).

##### 1.3.4.3 Biosensors and diagnostic devices

Another important application of conducting polymers is in biosensors, where their electrical properties are utilized to detect biological signals, such as pH changes, ion concentrations, or the presence of specific biomolecules. Conducting polymer-based sensors have been developed for monitoring glucose levels, detecting pathogens, and tracking cellular responses, making them valuable tools in medical diagnostics (Bhattacharyya, 2024).

## 2 Conclusion

A simple and direct method for producing biodegradable conducting polymer-based composites involves the direct mixing of a conductive polymer with biodegradable polymers. One key advantage of this approach is that the degradation rate and conductivity of the resulting composites can be tailored for specific biomedical applications by selecting the appropriate types and ratios of polymers to be blended. The flexibility of the direct blending method enables the combination of various conducting and biodegradable polymers, facilitating the development of numerous biodegradables conducting polymer-based scaffolds. These polymeric composites have been explored for a wide range of biological applications, particularly in tissue engineering and regenerative medicine. This technique effectively combines the strengths and beneficial properties of both types of polymers. However, it is important to note that the resulting composite may not fully exhibit the maximum conductivity and biodegradability of its individual components. For instance, the amount of polypyrrole (PPy) included in polymer blends is typically minimized to preserve the overall biodegradability of the composite, given the non-biodegradable nature of PPy. Consequently, while the copolymers may remain biodegradable, they may lack sufficient electrical conductivity for certain biological applications.
